# The *Bacillus subtilis yqgC-sodA* operon protects magnesium-dependent enzymes by supporting manganese efflux

**DOI:** 10.1128/jb.00052-24

**Published:** 2024-05-31

**Authors:** Ankita J. Sachla, Vijay Soni, Miguel Piñeros, Yuanchan Luo, Janice J. Im, Kyu Y. Rhee, John D. Helmann

**Affiliations:** 1 Department of Microbiology, Cornell University, Ithaca, New York, USA; 2 Division of Infectious Diseases, Weill Department of Medicine, Weill Cornell Medicine, New York, New York, USA; 3 School of Integrative Plant Sciences, Plant Biology Section, Cornell University, Ithaca, New York, USA; 4 Robert W. Holley Center for Agriculture and Health, USDA-ARS, Ithaca, New York, USA; 5 State Key Laboratory of Bioreactor Engineering, East China University of Science and Technology, Shanghai, China; The Ohio State University, Columbus, Ohio, USA

**Keywords:** superoxide dismutase, manganese, magnesium, metal homeostasis

## Abstract

Microbes encounter a myriad of stresses during their life cycle. Dysregulation of metal ion homeostasis is increasingly recognized as a key factor in host–microbe interactions. Bacterial metal ion homeostasis is tightly regulated by dedicated metalloregulators that control uptake, sequestration, trafficking, and efflux. Here, we demonstrate that deletion of the *Bacillus subtilis yqgC-sodA* (YS) complex operon, but not deletion of the individual genes, causes hypersensitivity to manganese (Mn). YqgC is an integral membrane protein of unknown function, and SodA is a Mn-dependent superoxide dismutase (MnSOD). The YS strain has reduced expression of two Mn efflux proteins, MneP and MneS, consistent with the observed Mn sensitivity. The YS strain accumulated high levels of Mn, had increased reactive radical species (RRS), and had broad metabolic alterations that can be partially explained by the inhibition of Mg-dependent enzymes. Although the YS operon deletion strain and an efflux-deficient *mneP mneS* double mutant both accumulate Mn and have similar metabolic perturbations, they also display phenotypic differences. Several mutations that suppressed Mn intoxication of the *mneP mneS* efflux mutant did not benefit the YS mutant. Further, Mn intoxication in the YS mutant, but not the *mneP mneS* strain, was alleviated by expression of Mg-dependent, chorismate-utilizing enzymes of the menaquinone, siderophore, and tryptophan (MST) family. Therefore, despite their phenotypic similarities, the Mn sensitivity in the *mneP mneS* and the YS deletion mutants results from distinct enzymatic vulnerabilities.

**IMPORTANCE:**

Bacteria require multiple trace metal ions for survival. Metal homeostasis relies on the tightly regulated expression of metal uptake, storage, and efflux proteins. Metal intoxication occurs when metal homeostasis is perturbed and often results from enzyme mis-metalation. In *Bacillus subtilis*, Mn-dependent superoxide dismutase (MnSOD) is the most abundant Mn-containing protein and is important for oxidative stress resistance. Here, we report novel roles for MnSOD and a co-regulated membrane protein, YqgC, in Mn homeostasis. Loss of both MnSOD and YqgC (but not the individual proteins) prevents the efficient expression of Mn efflux proteins and leads to a large-scale perturbation of the metabolome due to inhibition of Mg-dependent enzymes, including key chorismate-utilizing MST (menaquinone, siderophore, and tryptophan) family enzymes.

## INTRODUCTION

Metal ion homeostasis relies on a careful balance between metal import, export, and storage mechanisms (1). Bacterial growth may be restricted due to either metal ion limitation or excess, and both mechanisms are important during host–microbe interactions (2, 3). Nutritional immunity refers to the ability of the mammalian host to restrict access to essential nutrient metal ions such as iron (Fe), zinc (Zn), or manganese (Mn) (2). The host may also restrict the growth of intracellular pathogens through metal intoxication (3–5). In response, bacteria induce the expression of metal exporters that contribute to survival in the host (6, 7).

Metal ion efflux systems are regulated in opposition to metal importers. In *Bacillus subtilis,* the MntR metalloregulatory protein binds to Mn to repress the expression of the MntH and MntABCD importers (8). As Mn levels rise further, MntR activates the transcription of the MneP and MneS cation diffusion facilitator (CDF) efflux proteins (9). Although *B. subtilis* normally tolerates up to 1 mM Mn ion, mutants deficient in Mn homeostasis are much more sensitive (9, 10).

Previously, we investigated Mn intoxication using an *mneP mneS* (PS) efflux-deficient strain (11). When grown in minimal medium (MM), the Mn sensitivity of the PS strain can be suppressed by loss of function mutations in *qoxA* (part of the major aerobic respiratory quinol oxidase, QoxABCD) or *mhqR* (repressor of the methylhydroquinone-induced genes) (11). MhqR-regulated proteins function to reduce or degrade quinones and other reactive species (12, 13). Mn intoxication was thereby associated with production of reactive radical species (RRS) due to a dysfunction of the quinol oxidase (Qox) (cytochrome *aa*_3_) (11).

For reasons not yet understood, genetic suppression of Mn sensitivity is often dependent on the growth medium. For example, in MM with malate as a carbon source, but not with glucose, the Mn sensitivity of the PS mutant was suppressed by an insertion within the *yqgC-sodA* (YS) operon (*iTn-sodA*) (11). Conversely, in MM with glucose, but not with malate, the Mn sensitivity of the PS mutant was suppressed by the inactivation of *mpfA* (11), which encodes a Mg efflux pump (14). Inactivation of *mpfA* leads to increased intracellular Mg levels, suggesting that Mn intoxication can result from competition with Mg (14). Although Mg is the most abundant divalent cation in cells, it binds macromolecules less tightly than those ions in the Irving-William series: [Mn(II) < Fe(II) < Co(II) < Ni(II) < Cu(II) > Zn(II)]. Thus, Mg is susceptible to replacement by metals with higher affinities toward protein ligands (including Fe, Mn, and Zn).

Here, we describe the importance of the YS complex operon in Mn homeostasis. A strain with a deletion of the YS operon (YS mutant) had significantly reduced levels of *mneP* and *mneS* mRNA and is as sensitive to Mn as a PS efflux-deficient strain. Both strains accumulated high levels of intracellular Mn, displayed high levels of RRS, and had similar alterations in their metabolic profiles. Despite these similarities, the basis for Mn intoxication appeared to be distinct. Several mutations that suppressed Mn sensitivity of the PS mutant had little benefit for the YS strain. Conversely, the YS strain, but not the PS strain, was rescued by elevated expression of chorismate-utilizing, Mg-dependent enzymes of the menaquinone, siderophore, and tryptophan (MST) family. These findings reveal that Mn excess leads to specific metabolic disruptions, and the consequences of these disruptions can vary between phenotypically similar strains.

## RESULTS

### Mutants deleted for the YS operon are hypersensitive to Mn

**FIG 1:**
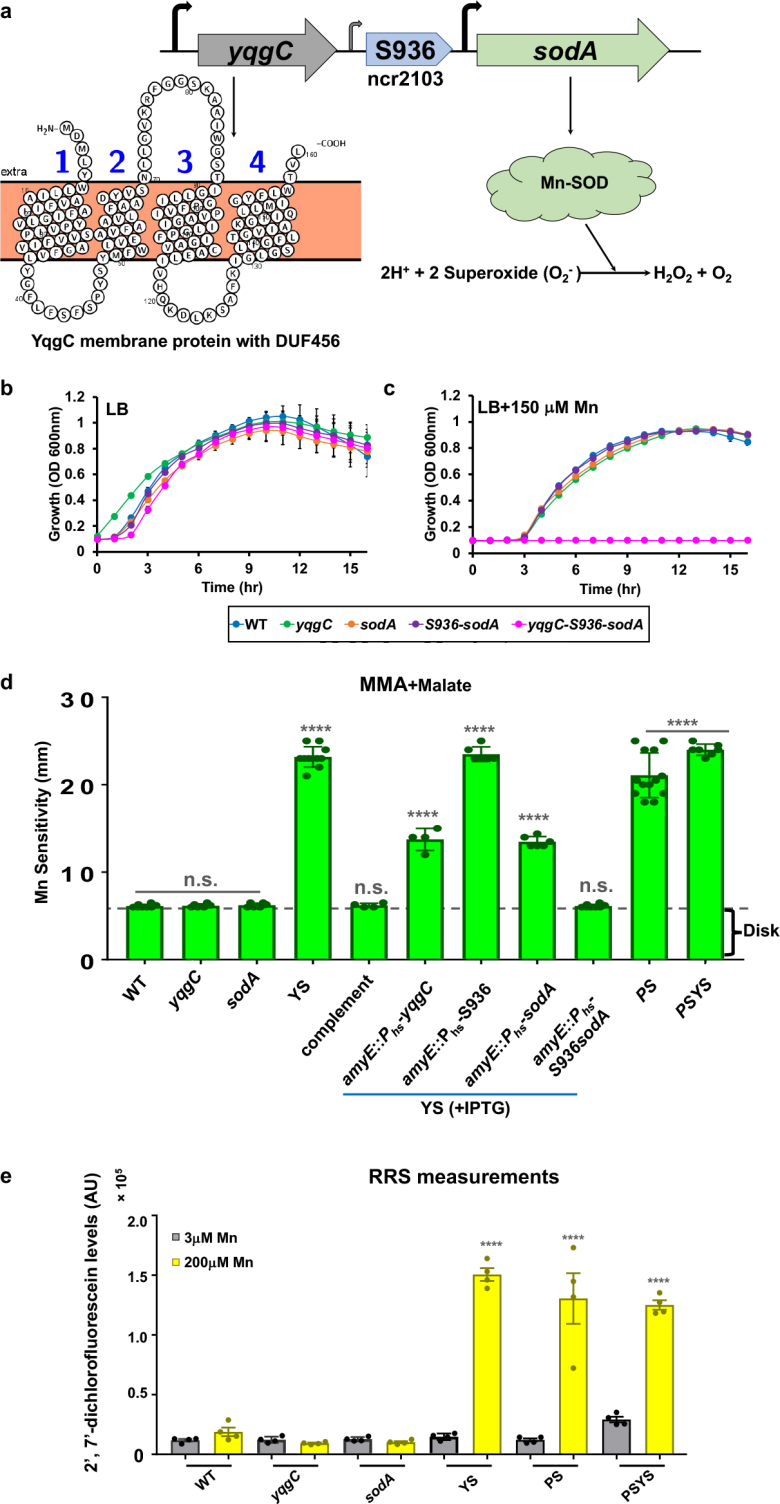
A YS operon deletion strain is hyper-sensitive to Mn. (a) The *yqgC-S936-sodA* (YS) complex operon is expressed from multiple promoters (bent arrows) and encodes: (i) YqgC, a 160 amino acids DUF456 membrane protein [lower left; illustrated using Protter (19)], (ii) a 178 bp intergenic region with a σ^B^ promoter (small arrow) that transcribes the S936 small RNA, also annotated as non-coding region ncr2103 (18), and (iii) SodA, a cytosolic MnSOD. Growth characteristics in (b) LB and (c) LB with 150 μM Mn at 37°C monitored in a plate reader illustrating the high Mn sensitivity of the *yqgC-S936-sodA* (YS) deletion strain. (d) Disk diffusion assay of Mn sensitivity. The diameter of clearance is shown around a Mn saturated disk (10 μL of 100 μM Mn) on minimal medium agar (MMA) plates with 0.8% malate (MMA+Malate) The dashed line is the disk diameter. Statistical analysis of 13 biological replicates compared to WT: ****P < 0.0001 using a one-way ANOVA test with Tukey’s post hoc analysis; n.s. no significant difference. (e) Aerobically grown, mid-logarithmic phase cells were resuspended in MM+malate with either low (3 μM) or high (200 μM) Mn and RRS formation was monitored with DCFDA at 37°C with shaking in a plate reader. Fluorescence emission normalized to culture optical density (OD) is tabulated in arbitrary units (AU). RRS production in high Mn was compared to the corresponding low Mn condition for each strain using a one-way ANOVA test with Tukey’s post hoc analysis (*n* = 4: ****P < 0.0001).

Manganese-dependent superoxide dismutase (MnSOD) is one of the most abundant proteins in the *Bacillus* proteome (15) and is the major Mn-binding protein in the cell (16). The *sodA* gene is transcribed together with the upstream *yqgC* gene as part of a complex, two-gene operon, YS (Fig. 1a). YqgC encodes an unknown function (DUF456) membrane protein of 160 amino acids. Transcription initiates upstream of *yqgC* and from two promoters within the 178 bp YS intergenic region (17), with the upstream intergenic promoter associated with the production of a 128 nt long 5′-untranslated region designated ncr2103 (18) or S936 (17) (Fig. 1a).

Previously, we noted that Mn intoxication can lead to an accumulation of dichlorodihydrofluorescein (DCF)-RRS (11), suggesting that MnSOD may be a protective protein under conditions of Mn intoxication. To explore the role of the YS operon in Mn homeostasis, we generated in-frame deletions of the *yqgC* and *sodA* coding regions using the BKE collection of mutant strains (20). In these strains, the coding region is replaced by an erythromycin cassette that can be removed using plasmid pDR244 expressing the Cre-Lox recombinase (20). In addition, we used a CRISPR-based mutagenesis approach (21) to delete just the S936 element, the S936-*sodA* region, or the YS operon in its entirety (*ΔyqgC*-S936-*sodA;* YS). The *yqgC*, *sodA*, and S936-*sodA* deletion strains all grew well in Luria-Bertani (LB) medium both with and without amendment with 150 μM Mn (Fig. 1b and c). However, the YS operon deletion was unable to grow in LB medium amended with 150 μM Mn (Fig. 1c).

We next quantified Mn sensitivity on MM agar plates with malate (MMA+malate). The YS strain exhibited a large clearance zone equivalent to the Mn-sensitive *mneP mneS* efflux knockout strain (Fig. 1d). This zone of clearance was not observed in the *yqgC* and *sodA* single mutants. Although both the YS and PS mutants are Mn sensitive, a quadruple mutant (PSYS) was no more sensitive than the YS or PS double mutants (Fig. 1d). Thus, cells lacking the entire YS operon are highly Mn sensitive. This high sensitivity was also seen in cells grown in liquid cultures: the MIC for Mn in MM-malate revealed a 400-fold increase in sensitivity in the YS strain (MIC of 5 μM) compared with the wild-type (WT) or single deletions strains (MIC of 2 mM) (Fig. S1). We conclude that the YS operon is important for WT levels of resistance to elevated Mn, but loss of either *yqgC* or *sodA* is well tolerated.

To better understand the contributions of *yqgC *and *sodA* genes and the S936 untranslated region to Mn resistance, we expressed these elements from an ectopic site in the YS mutant strain. We were able to partially complement YS Mn sensitivity by expressing either *yqgC* or *sodA* at *amyE* locus using the P*_hyperspank_* (P_hs_) promoter with isopropyl β-D-1-thiogalactopyranoside (IPTG) as inducer. Further, YS Mn sensitivity was completely abolished by expression of either the S936-*sodA* region or the entire *yqgC*-S936-*sodA* operon (Fig. 1d).

### Mn intoxication has similar consequences in YS and PS

Since the YS mutant was as Mn sensitive as the efflux-deficient PS mutant (Fig. 1d), we compared these two strains by monitoring Mn accumulation, production of RRS, and metabolome profiles. When grown in LB medium and challenged with 150 μM Mn, the intracellular level of Mn increased in all strains, but most dramatically in the YS and PS mutant strains (Table 1). After Mn challenge, the YS strain accumulated ~15× the level of Mn seen in the WT strain, and the PSYS strain accumulated ~7 fold more (Table 1). In contrast, Mn accumulation was unaffected by the individual deletion of either *yqgC* or *sodA* (Table 1). This large change in intracellular metal content was specific to Mn, and other ions (Zn, K) were little affected. However, elevated Mn was correlated with a decrease in Mg in some strains (Table 1).

**TABLE 1:** Intracellular metal levels (μg g^−1^ cells dry weight)^*c*^

Strains	Mn levels		Mg levels		K levels		Zn levels	
	LB	LB + Mn	LB	LB + Mn	LB	LB + Mn	LB	LB + Mn
WT	0.6 ± 0.05	3.2 ± 1.3	46 ± 3	43 ± 0.4	3,660 ± 210	5,102 ± 90	1.3 ± 0.15	1.4 ± 0.1
*yqgC*	0.4 ± 0.1	2.5 ± 0.8	26.5 ± 5	34 ± 2	3,271 ± 175	5,027 ± 398	1.3 ± 0.6	1 ± 0.1
*sodA*	0.4 ± 0.16	3.3 ± 1.9	33 ± 12	38 ± 3.7	3,320 ± 59	5,111 ± 78	1.3 ± 0.4	1.4 ± 0.47
S936	0.6 ± 0.3	3.3 ± 1.5	38 ± 12	44 ± 10	3,245 ± 72	5,139 ± 122	1.8 ± 0.5	1 ± 0.7
S936-*sodA*	0.6 ± 0.33	6 ± 0.4	49 ± 3.4	49 ± 4	3,378 ± 7	5,094 ± 366	1.8 ± 0.5	1.5 ± 0.4
YS	0.5 ± 0.2	**47 ± 7.2^*a*^**	38 ± 5.5	**19 ± 2^*b*^**	3,168 ± 318	4,708 ± 100	2 ± 0.14	1.17 ± 0.05
PSYS	0.5 ± 0.05	**22 ± 1.8^*b*^**	23 ± 1.2	**10 ± 1.6^*a*^**	3,112 ± 320	4,842 ± 41	1.3 ± 0.3	0.9 ± 0.25
YS *meeF-*I206T	0.5 ± 0.048	**28 ± 13^*a*^**	34 ± 7	**18 ± 6^*a*^**	3,119 ± 97	4,951 ± 253	2.2 ± 0.4	1.1 ± 0.05
YS *meeF-*F225V	0.4 ± 0.07	5.5 ± 1.3	35 ± 8	33 ± 4.3	3,690 ± 124	4,890 ± 232	1.8 ± 1	1.2 ± 0.25
YS *meeY-*W71R	0.5 ± 0.03	**51 ± 9^*a*^**	38 ± 3.2	**23 ± 2.4^*b*^**	3,253 ± 118	4,917 ± 242	2.4 ± 0.7	1.0 ± 0.05
YS mTn *yazB*	0.5 ± 0.047	**22 ± 3^*a*^**	25 ± 6.3	**12 ± 6.5^*a*^**	2,934 ± 184	4,661 ± 626	1 ± 0.2	1 ± 0.25
PS	0.5 ± 0.1	**34 ± 12^*a*^**	36 ± 9	**18 ± 5^*b*^**	3,413 ± 453	3,571 ± 442	1.8 ± 0.2	2 ± 0.8
*mneP*	0.8 ± 0.03	4 ± 0.6	47 ± 3	47 ± 3.3	3,162 ± 56	3,302 ± 180	2 ± 0.3	2.2 ± 0.5
*mneS*	0.8 ± 0.05	3 ± 0.7	43 ± 5	35 ± 2.3	3,234 ± 192	2,991 ± 81	1.7 ± 0.25	1.6 ± 0.5

^*a*^ *P* < 0.0001 (bold text).

^*b*^ *P* < 0.001, but >0.0001 (bold text).

^*c*^ All comparisons were made with WT either treated or untreated for the same ion using a one way ANOVA with Tukeys post-test.

Previously, the PS efflux-deficient strain was found to accumulate RRS when challenged with Mn (11). These studies monitored RRS using the fluorogenic reporter 2′,7′-DCF diacetate, which is de-acetylated in cells to generate DCF. DCF is readily oxidized by one-electron oxidizing species, such as hydroxyl radical and other reactive radicals generated by the Fenton reaction (22, 23). Like the PS strain, the YS mutant and the PSYS mutant also accumulated high levels of RRS after Mn challenge (Fig. 1e). We conclude that the YS strain, like the PS strain, accumulates Mn intracellularly and experiences increased stress from RRS.

The nature of the RRS detected by DCF are not entirely clear, but DCF can report on hydroxyl radicals, such as those generated by Fe-dependent Fenton chemistry. As Fenton chemistry is driven by hydrogen peroxide (H_2_O_2_), and MnSOD generates H_2_O_2_ from superoxide, we tested the role of the YS operon in intrinsic resistance to the superoxide generator paraquat (Fig. S2). As expected, mutants lacking *sodA* were more sensitive to paraquat (MIC 4 μM) than WT (MIC 60 μM). In addition, the *yqgC* mutant displayed an intermediate phenotype (MIC 15 μM). There was no apparent additivity seen in YS compared to *sodA* alone, which indicates that MnSOD is the key protective factor and that YqgC may play a supportive role in MnSOD function (Fig. S2).

### Mn sensitivity in the YS strain is correlated with defects in Mn efflux

**FIG 2:**
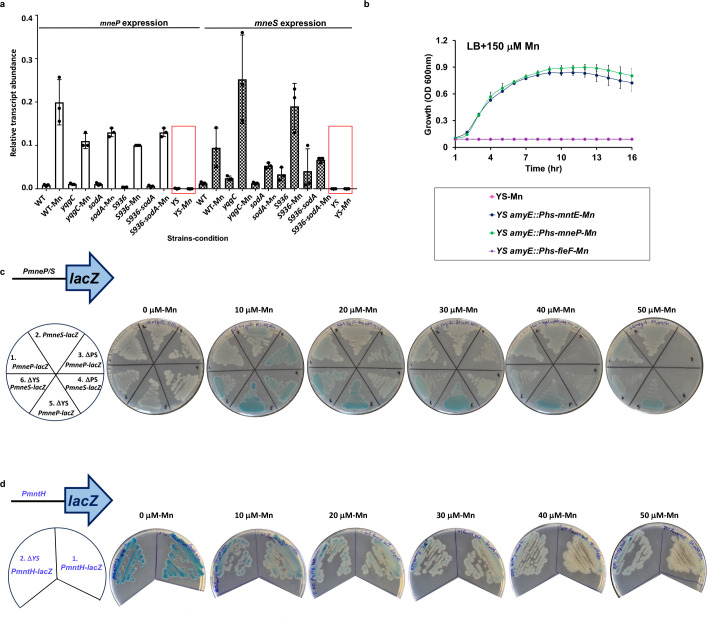
The YS deletion has reduced transcript levels for the *mneP* and mneS efflux pumps. (**a**) Transcript levels of the Mn-inducible *mneP* (left panels, no fill) and *mneS* (right panels, hatched bars) genes were monitored in the indicated strains (WT, *ΔyqgC*, *ΔsodA*, *ΔS936*, *ΔS936-sodA,* and YS) after growth in LB till OD_600nm_ = 0.4 with and without a 150 μM Mn shock for 30 min. RNA was purified and expression levels determined by qRT-PCR and normalized to *gyrA*. Values shown are mean ± SD from three independent experiments. Red boxes are shown to emphasize the loss of *mneP* and *mneS* transcripts in YS with Mn. (**b**) Growth in LB+0.2 mM IPTG at 37°C with 150 μM Mn demonstrates that expression of Mn efflux proteins (P*_hs_-mneP*, *P_hs_-mntE),* but not an Fe efflux protein (*P_hs_-fieF*; overlaps with Y alone) can rescue growth of the Mn-sensitive YS strain. (**c**) The YS strain is competent for MntR-dependent activation of Mn-regulated P*_mneP_* (odd numbers) and P*_mneS_* (even numbers) *lacZ* fusions, as seen also in WT and PS strains. Note: PS and YS are hypersensitive to Mn and at higher levels show plating defects. (**d**) The YS strain is competent for the MntR-dependent repression of the MntR-regulated P*_mntH_* promoter.

We hypothesized that the Mn sensitivity of the YS strain may result from a defect in the expression or function of the MneP and MneS efflux pumps. We, therefore, monitored the levels of *mneP* and *mneS* mRNA using quantitative real-time reverse transcription PCR (qRT-PCR) (Fig. 2a). In WT, the *mneP* transcript was induced by challenge of cells to 150 μM Mn for 30 min., and a similar response was seen for the three single mutants (*yqgC,* S936*,* and *sodA*), and the S936-*sodA* mutant. However, mRNA levels were reduced and no longer inducible in the YS mutant (Fig. 2a). A similar effect was noted for the *mneS* mRNA, although the response to Mn was more variable between strains (Fig. 2a). These observations suggest that the YS operon deletion reduces the expression of the MneP and MneS efflux proteins.

We next tested whether the Mn sensitivity of the YS mutant could be rescued by overexpression of MneP from an IPTG-inducible promoter. Indeed, induction of MneP restored the ability of YS to grow in LB medium amended with 150 μM Mn (Fig. 2b). Similarly, expression of MntE, a *Staphylococcus aureus* CDF (24) that is a homolog of *B. subtilis* MneS (41% identity) and MneP (26% identity), also restored Mn resistance (Fig. 2b). In contrast, expression of the *Escherichia coli* FieF CDF protein (22% identity to MneP, 21% identity to MneS), implicated in Zn, Fe, and Mn efflux (25, 26), did not restore Mn resistance to the *B. subtilis* YS mutant (Fig. 2b). These results support the hypothesis that Mn sensitivity in the YS strain results from a deficiency in Mn efflux.

Transcriptional induction of *mneP* and *mneS* by excess Mn is dependent on the MntR (manganese transcriptional regulator) transcription factor (9). We hypothesized that the lack of *mneP* and *mneS* mRNA accumulation might result from a failure of MntR to activate transcription. However, when we monitored the induction of P*_mneP_-lacZ* and P*_mneS_-lacZ* promoter-*lacZ* fusions by Mn both genes were strongly induced in the YS strains, comparable to that seen in the PS strain. The stronger induction in the YS and PS strains compared to WT seen with 10 μM Mn (Fig. 2c) is consistent with expected higher accumulation of Mn in the YS and PS strains. Similarly, the ability of MntR to function as a Mn-dependent repressor, as monitored using a P*_mntH_-cat-lacZ* reporter fusion, was unimpaired (Fig. 2d). We, therefore, hypothesize that the reduced levels of *mneP* and *mneS* mRNA in the YS strain, as seen in LB medium with and without Mn shock (Fig. 2a), may result from a reduction in mRNA synthesis or stability at a step after the MntR-dependent initiation of transcription. The nature of this effect is presently unclear.

### Genetic suppression of Mn sensitivity in the YS mutant

**FIG 3:**
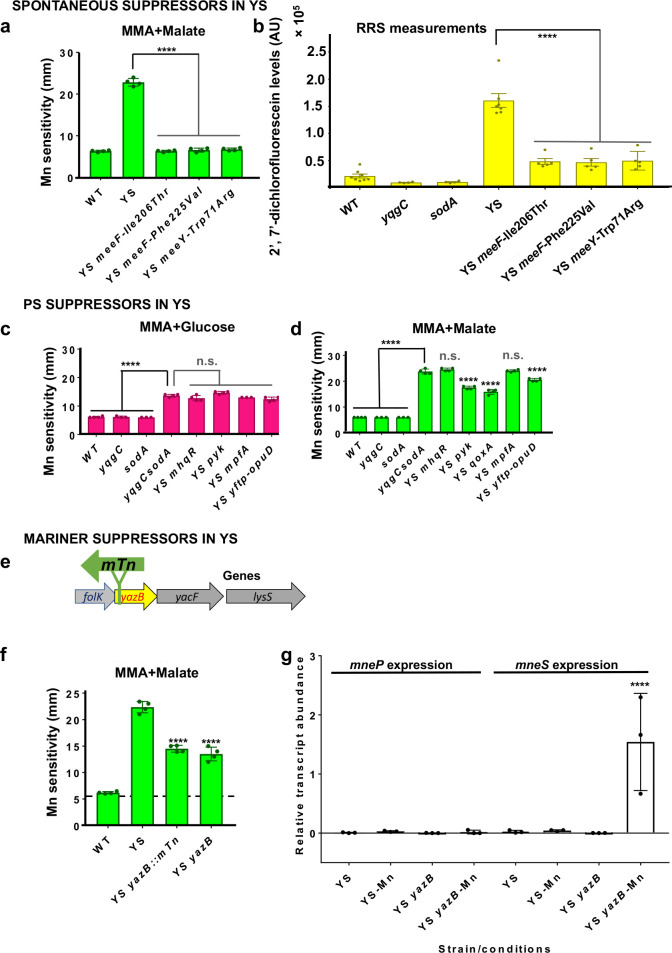
Genetic identification of genes affecting Mn resistance in YS. (a) Spontaneous suppressors of Mn sensitivity were recovered from the zone of inhibition in the YS background. Whole-genome re-sequencing (Table S1C) identified point mutations in the *meeF* and *meeY* genes encoding TerC proteins associated with Mn metalation of exoenzymes (27, 28). The mutations were re-constructed (using CRISPR) into the YS strain and the Mn-resistant phenotype tested by disk diffusion on MMA+Malate plates. (b) The same mutations also reduce the level of Mn-stimulated RRS production in MM+200 μM Mn as monitored using DCFDA. (c and d) Mutations that suppress Mn sensitivity of the PS efflux-deficient strain (*mhqR*, *pyk*, *mpfA*, *qoxA*, and *ytfP-mTn-opuD*) (11) do not suppress YS. Mutations were introduced into YS and Mn sensitivity was determined by measuring the zone of clearance (diameter, mm, inclusive of the 6.5 mm disk) after 18 h incubation on MMA at 37°C with (c) glucose or (d) malate (*n* = 4 and ****adjusted *P* < 0.0001 using a one-way ANOVA test with Tukey’s post hoc analysis; n.s. no significant difference compared to YS). (e and f) *yazB*::mTn insertion partially suppresses Mn sensitivity of the YS mutant as monitored by the zone of clearance (diameter, mm, inclusive of the 6.5 mm disk) after 18 h incubation on MMA+malate at 37°C. *n* = 4; ****adjusted *P* < 0.0001 using a one-way ANOVA test with Tukey’s post hoc analysis relative to YS. (g) Analysis of transcript levels of *mneP* and *mneS* by qRT-PCR (normalized to *gyrA*) in the YS and YS *yazB* strains grown in LB till OD_600nm_ = 0.4 and then amended with or without 150 μM Mn for 30 min. Values are mean ± SD from three independent experiments.

To better understand the effects of Mn on the YS strain, we selected spontaneous mutants that formed colonies in the zone of clearance around Mn disks in zone of inhibition assays. Results from whole genome-resequencing (Table S1C) revealed that these isolates often carried missense mutations affecting either of two TerC homologs (MeeF and MeeY) implicated in Mn export and exoprotein metalation (27, 28). As expected based on prior work (27), null mutations in *meeF* or *meeY* did not increase Mn resistance in the YS background. Therefore, these missense mutations were recreated by CRISPR-based genome editing to test whether their introduction was sufficient for Mn resistance. In each case, CRISPR-generated strains with only the altered function meeF* or meeY* alleles were Mn resistant (Fig. 3a), and they also greatly reduced the ability of Mn to induce RRS production (Fig. 3b). As TerC homologs are implicated in Mn efflux (27, 28), we used inductively coupled argon plasma-optical emission spectrometry (ICP-ES) to measure the impact of meeF* or meeY* alleles on Mn levels. Our ICP-ES results suggest that the meeF* allele (Phe225Val) decreased Mn accumulation in the YS cells to near WT levels, likely accounting for the increased Mn resistance (Table 1). In contrast, a more modest reduction was noted in the YS meeF* (Ile206Thr) strain, and no reduction of Mn was observed in the YS meeY* (Trp71Arg) strain (Table 1). Thus, these mutations may lead to Mn sequestration or other changes in Mn pools that thereby reduce RRS generation.

Since the YS and PS strains have similar levels of Mn accumulation and RRS production, we anticipated that suppressor mutations previously shown to rescue Mn sensitivity of the PS strain (11) would also rescue the YS strain. Unexpectedly, none of the mutations that improved fitness of the PS mutant strain on MM+glucose (*mhqR*, *pyk*, *mpfA*, *ytfP-opuD*) were able to improve the growth of the YS strain on this medium (Fig. 3c). The mutation that most strongly rescued growth of the PS strain on MM+malate (*qoxA*) had a modest beneficial effect in the YS strain (Fig. 3d). A small beneficial effect was also noted for the *pyk* mutation in YS (Fig. 3d), even though this mutation was not beneficial to the PS strain on this medium [and was selected on MMA+glucose (11)]. While the basis for these genetic interactions is poorly understood, these differences imply that, despite their phenotypic similarities, the processes that limit growth in the PS and YS strains may be different.

Next, we used mariner transposon (*mTn*) mutagenesis to isolate mutations that could suppress Mn sensitivity of the YS strain. We recovered eight different transposants with insertions in a variety of loci (Table S1D), and all of which were genetically linked to the observed Mn resistance. We here focused on strain HBYL1110 with an *mTn* insertion after position 87484 in the *Bacillus* reference genome (NC_000964 (29); within *yazB*. The *yazB* gene is part of a complex operon including genes for folate (PabB) biosynthesis (*folB-folK-yazB-yacF-lysS*) (Fig. 3e). YazB is predicted to be a small (69 amino acid) DNA-binding protein, suggestive of a possible regulatory function. Prior studies of a *yazB* mutant did not reveal changes in the expression of the upstream PabB genes (30). We confirmed that an in-frame *yazB* deletion mutant suppressed Mn sensitivity of the YS strain (Fig. 3f).

As YS strains are defective in the expression of *mneP* and *mneS*, we monitored the effect of the *yazB* on these two genes. Remarkably, Mn-dependent induction of *mneS* was restored in the YS *yazB* strain, but expression of *mneP* expression was still non-responsive to Mn stress (Fig. 3g). This suggests that the *yazB* suppressor works by restoring the expression of *mneS*, consistent with the conclusion above that YS fails to properly express the MneP or MneS efflux proteins. We attempted to test this hypothesis by constructing a YS *yazB mneS* quadruple mutant, but were repeatedly unable to obtain this strain.

### Mn intoxication elicits similar changes in the metabolome of YS and PS strains

Next, we used metabolomics to monitor the global changes in metabolism upon challenge of WT, *yqgC*, *sodA*, and YS strains grown in LB with and without 150 μM Mn and compared these changes to those seen in the efflux-deficient PS mutant. The *yqgC* and *sodA* single mutants had relatively modest metabolic perturbations after Mn challenge. In contrast, the Mn-sensitive YS and PS strains had similar and wide-ranging changes in cellular metabolism (Fig. S3).

**FIG 4:**
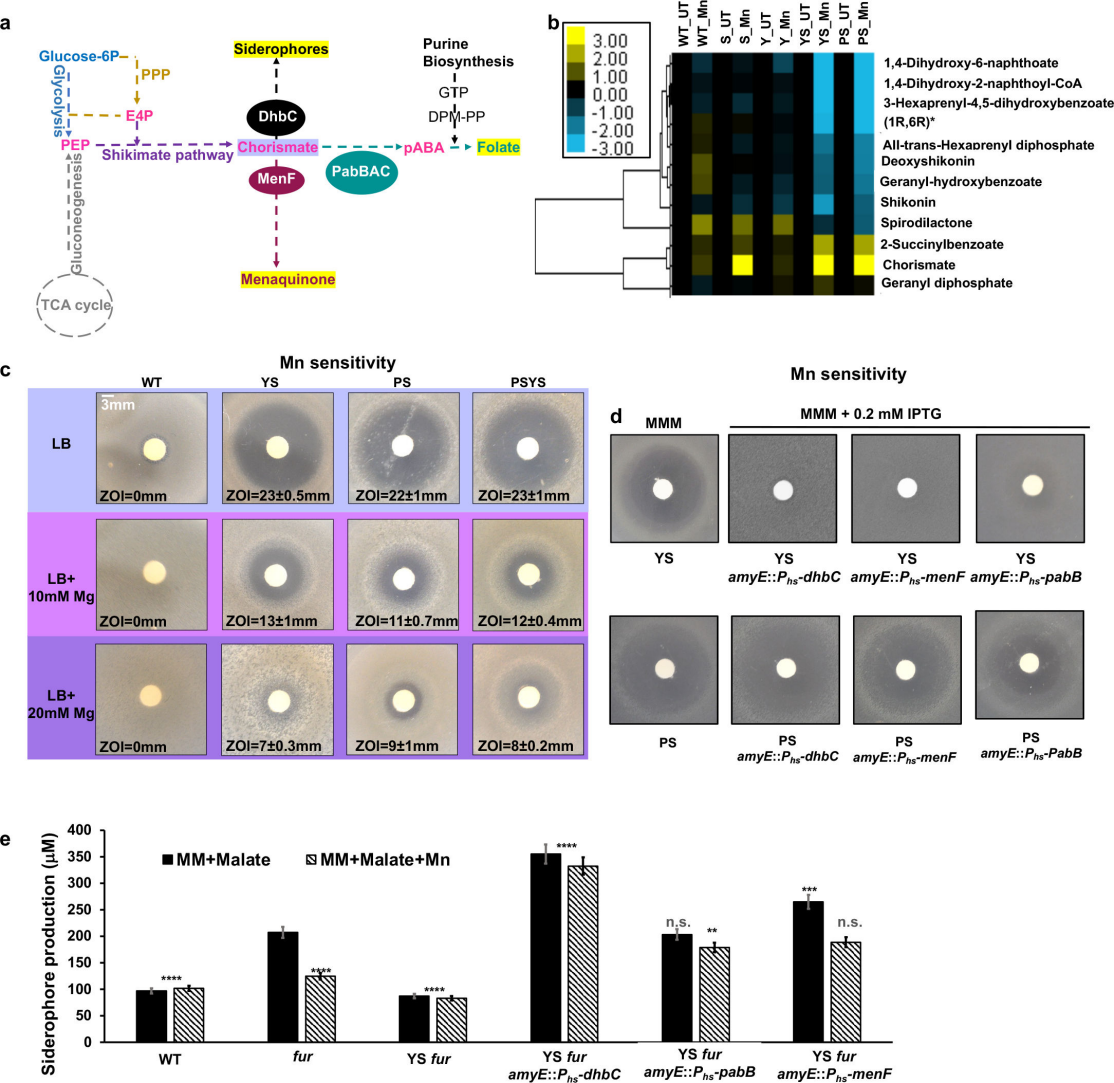
Chorismate-dependent metabolites are affected in the absence of YS. (**a**) Schematic showing the role of chorismate as a branch-point metabolite. Phosphoenolpyruvate (PEP) is generated from glycolysis (blue) and gluconeogenesis (grey) and erythrose-4-phosphate (E4P) is generated by the pentose-phosphate pathway (PPP, orange). PEP and E4P are funneled into the shikimate pathway (purple) to generate chorismate, a precursor for other metabolites including menaquinone, siderophores, and PabB as shown. (**b**) Selected metabolites of the shikimate, PabB, and menaquinone pathways are shown for the *yqgC* (Y), *sodA* (S), YS, and PS strains (see also Fig. S2). Color bars represent log_2_ fold changes. (**c**) Mn sensitivity of the YS, PS, and PSYS strains can be suppressed by elevated Mg as measured by disk diffusion assay on LB medium. (**d**) Mn sensitivity of the YS, but not the PS, strain can be suppressed by induction of selected MST family enzymes (*menF*, *dhbC*, and *pabB*) as monitored on MMA+malate+0.2 mM IPTG. (**e**) DHBA-based siderophore yield (absorbance 510 nm/OD_600nm_) was determined with and without over-expression of MST enzymes (*menF*, *dhbC*, and *pabB*). Cells were grown overnight in MM+malate with 1 μM ferric ammonium citrate and 0.2 mM IPTG for strains with inducible constructs. Mid-log phase cells were resuspended in the same medium with no iron and growth continued for 5 h. was used in the determination of DHBA levels after addition of excess FeCl_3_. The level of siderophore in the supernatant was calculated from a DHBA standard curve (*n* = 2: the difference in siderophore production among all strains was compared to the *fur* mutant grown in MM+malate using a one-way ANOVA test with Tukey’s post-hoc analysis where ****P < 0.0001, ***P ≤ 0.001, and **P < 0.01, with n.s. no significant difference (when compared with *fur::kan*).

Inspection of the metabolomics results revealed striking effects on chorismate-dependent pathways (Fig. 4a). Specifically, there was an accumulation of chorismate upon Mn-treatment of the *sodA* mutant, as well as the YS and PS strains (Fig. 4b). In addition to an elevation of chorismate, the YS and PS strains also displayed a Mn-dependent decrease in downstream metabolites, including the tryptophan precursor anthranilate and the menaquinone precursors 1,4-dihydroxy-2-naphthoic acid (DHNA) and (1R,6R)−6-hydroxy-2-succinyl-cyclohexa-2,4-diene-1-carboxylate (Fig. 4b; Fig. S3).

These metabolic perturbations led us to hypothesize that Mn might inhibit enzymes of the MST family (Fig. 4a). *B. subtilis* encodes several MST-family enzymes involved in the synthesis of the bacillibactin siderophore (DhbC), PabB, and menaquinone (MenF). MST enzymes bind chorismate, with the carboxylate group coordinated to divalent magnesium (Mg^2+^), and catalyze a variety of addition reactions (with either water or ammonia as nucleophile) that may or may not be coupled to the elimination of pyruvate (31–33). The metabolomic data suggest that other Mg-dependent enzymes may also be inhibited under these conditions, including Mg-dependent amidotransferases involved in aromatic amino acid (Trp, Phe, Tyr) biosynthesis. In addition, asparagine levels are greatly reduced in the Mn-challenged YS and PS mutants (Fig. S3), and asparagine synthase is a Mg-dependent amidotransferase (34). Similarly, inhibition of the HisC/AroJ amidotransferase may account for the observed increase in phenylpyruvate and decrease in phenylalanine (Fig. S3).

A prior work demonstrates that ferrous Fe can displace Mg and thereby serve as a nanomolar inhibitor for multiple MST family enzymes, including isochorismate synthases (*Pseudomonas aeruginosa* PchA and *E. coli* EntC) and salicylate synthase (*Yersinia enterocolitica* Irp9) (33). We, therefore, hypothesized that Mn, previously shown to be inactive as a cofactor for isochorismate synthase (31), might serve as a general inhibitor of MST family enzymes in *B. subtilis*. To test this hypothesis, we asked whether supplemental Mg could suppress Mn sensitivity. Indeed, Mg reversed the Mn-dependent growth inhibition of the YS, PS, and PSYS strain on LB plates (Fig. 4c). Thus, Mn-dependent inhibition of Mg-dependent enzymes like contributes to the observed growth inhibition by elevated Mn.

### Induction of MST family enzymes reverses Mn intoxication of YS but not PS

To test the hypothesis that inhibition of Mg-dependent MST enzymes is limiting for growth, we engineered strains with inducible expression of several of these enzymes. Surprisingly, induction of any of the three MST proteins suppressed Mn intoxication of the YS strain (Fig. 4d) but not the PS strain in LB medium (Fig. 4d). These results suggest that Mn intoxication inhibits a similar set of enzymes in the PS and YS strains, but the specific metabolic deficiencies that underlie growth inhibition differ.

Of all the products of pathways dependent on MST enzymes, the simplest to assay is the siderophore bacillibactin and its precursors. In *B. subtilis* 168 strains, this pathway is not fully functional due to the presence of the *sfp^0^* mutation that inactivates the Sfp 4-phosphopantetheinyl transferase necessary for the activity of non-ribosomal peptide synthases (35, 36). As a result, 168 strains fail to properly modify the DhbB and DhbF enzymes with the phosphopantetheine carrier and produce the bacillibactin precursors 2,3-dihydroxybenzoic acid (DHBA) and 2,3-dihydroxybenzoylglycine (DHBG), known collectively as DHBA(G) (37). These precursors are easily assayed in cell supernatants of iron-starved cells using spectrophotometry. We monitored DHBA(G) production using *fur* mutants that derepress the expression of the entire *dhb* operon for bacillibactin production (38). The use of *fur* mutants also prevents any effect of Mn on Fur-mediated repression of the *dhb* operon as seen under some growth conditions (39). As expected, DHBA(G) production was elevated in cells grown in MM+malate in the *fur* mutant. However, DHBA(G) production was not elevated if the medium was amended with Mn, or in the YS *fur* strain (Fig. 4e; Fig. S4). Further, DHBA(G) production was increased even above the levels seen in the *fur* mutant in strains overexpressing the DhbC isochorismate synthase, and synthesis was no longer inhibited by Mn (Fig. 4e). DHBA(G) synthesis was also increased by expression of a different isochorismate synthase, MenF. These observations are consistent with the hypothesis that Mn is a generalized inhibitor of MST enzymes, and this inhibition can be overcome by Mg supplementation or by enzyme overproduction. Unexpectedly, the induction of *pabB* also increased the expression of DHBA(G) (Fig. 4e; Fig. S4). Although PabB is an MST-family enzyme, it is not known to have isochorismate synthase activity. Thus, the mechanism of this effect may be indirect, perhaps through the binding of Mn and protection of DhbC from Mn inhibition.

## DISCUSSION

Metalloenzymes are ubiquitous in biology, but their activity is contingent on metalation with the proper metal cofactor. Many metalloproteins obtain the cognate metal ion from a buffered cytosolic pool dependent on metal availability and the metal affinity of the protein active site (40). According to the Irving-Williams series, divalent transition metals typically bind with affinities in the order: Mn(II) < Fe(II) < Co(II) < Ni(II) < Cu(II) > Zn(II). These thermodynamic affinities often determine the hierarchy for metal occupancy within a specific metalloenzyme. The metalation of enzymes that require high-affinity metals, such as Cu or Zn, typically involves ligand exchange reactions with cellular metabolites or protein metallochaperones. For lower affinity metals, such as Mn and Fe, the buffered metal pools are more loosely held, and metal acquisition may occur unaided from pools of rapidly exchanging ions. In both cases, metalation by more tightly held and slower exchanging metals (from higher in the Irving–Williams series) can lead to enzyme mismetalation and inactivation. For example, Zn intoxication has been linked to the mismetalation of proteins that normally require Mn or Fe for their activity (41–43).

Even though Mn and Fe are relatively weak-binding metals (at the lower affinity end of the Irving–Williams series), cells can still experience metal intoxication when their homeostasis systems are disrupted. In *B. subtilis*, Fe intoxication is apparent in strains lacking the PfeT Fe efflux ATPase (44, 45), and Mn intoxication is apparent in efflux-deficient PS strains (9, 11) and, as shown here, in the absence of the YS operon. The molecular basis for intoxication by these more weakly interacting metal ions is not well understood, but previous results have suggested that excess Mn may interfere with Mg-dependent processes (14, 46). For example, mutations in a *B. subtilis* Mg-efflux system (MpfA) can increase tolerance to high Mn levels (14). However, the Mg-dependent pathways that are inhibited by Mn have not been defined. Our prior work reveals that Mn intoxication in PS derives, in part, from a dysfunction associated with the QoxABCD terminal oxidase and is correlated with production of RRS (11).

Here, we focused on understanding the role of the YS operon in Mn homeostasis. The YS operon deletion mutant is phenotypically similar to the PS strain characterized previously (11). Both the YS and PS mutants display similar Mn sensitivity (Fig. 1d), accumulation of intracellular Mn and a concomitant reduction in Mg when excess Mn is added (Table 1), Mn-induced production of RRS (Fig. 1e), and changes to their metabolomes (Fig. 4b; Fig. S3). In addition, the Mn sensitivity of both strains can be suppressed by high Mg (Fig. 4c). This similarity is supported by a lack of additivity between the PS and YS mutations (Fig. 1d), which is consistent with the observation that the YS strain is defective for expression of *mneP* and *mneS* (Fig. 2a), encoding two efflux proteins lacking in the PS strain. The mechanism underlying the reduced levels of *mneP* and *mneS* mRNAs in the YS mutant (Fig. 2a) is unclear but does not appear to be due to an inability of MntR to activate transcription (Fig. 2c).

The phenotypes reported here are most evident in strains lacking both *yqgC* and *sodA*, but not in the single mutant strains. Superoxide dismutase is a broadly conserved protein found in all domains of life (47). In *B. subtilis*, MnSOD is the major Mn-binding protein in the cell (16) and protects cells against the reactive superoxide radical. The co-transcribed *yqgC* gene encodes an unknown function (DUF456) membrane protein. YqgC and its homologs (COG2839) are present in >1,000 sequenced genomes, including *Deinococci*, *Mycobacteroides abscessus*, and *Salmonella enterica*. Although both *sodA* and *yqgC* are broadly conserved in many bacteria, gene neighborhood analysis reveals that the YS genomic organization is predominantly seen in the *Bacillus* genus and occasionally in other members of the *Bacillaceae*.

It is not obvious why the deletion of both genes is required to reveal a high level of Mn sensitivity. However, this sensitivity is correlated with RRS accumulation (Fig. 1d and e). Our prior work linked RRS production to a dysfunction of the major quinol oxidase (QoxA)-dependent electron transport chain (ETC) (11). One possibility is that the YqgC membrane protein interacts with proteins of the ETC or with MenF itself to help prevent RRS production, and in its absence MnSOD helps prevent RRS accumulation.

Both the YS and PS strains have similar metabolic profiles when challenged with Mn (Fig. S3). Several of the over-represented metabolites are substrates of Mg-dependent enzymes, consistent with the finding that elevated Mg suppresses intoxication (Fig. 4c). However, the proximal cause of growth inhibition in these two strains appears to be different. Overexpression of MST family enzymes rescued the YS strain, but not the PS strain (Fig. 4d). We therefore conclude that Mg-dependent MST family enzymes are inhibited in these strains, but other Mg-dependent processes also fail in the PS strain, precluding growth rescue by simply overexpressing MST enzymes.

**FIG 5:**
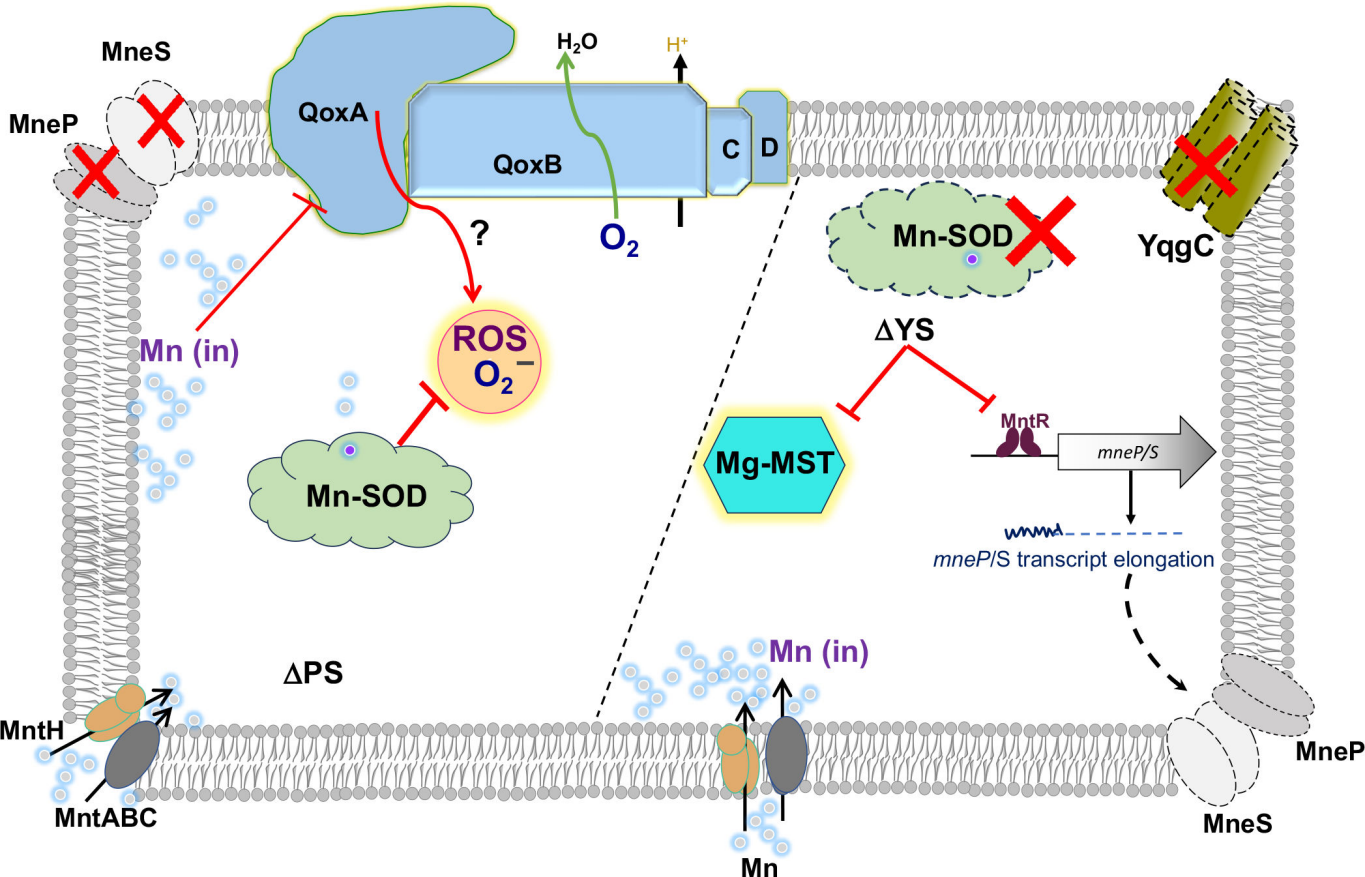
Functions of YS operon are needed for Mn homeostasis. In the absence of the MneP/S Mn efflux proteins (left panel) cells accumulate Mn, which causes the production of DCF-RRS, which may be generated from superoxide and other reactive oxygen species (ROS) emanating from the electron transport system. This stress is reduced by deletion of *qoxA*, a subunit of the Qox (11). Cytosolic superoxide is eliminated by Mn-SOD encoded by *sodA*. The *sodA* gene is transcribed with *yqgC* encoding a DUF456 membrane protein. In the YS deletion strain (right panel), transcript levels of *mneP* and *mneS* are significantly reduced (Fig. 2a) leading to Mn sensitivity, even though the ability of MntR to activate these promoters appears to be unaffected (Fig. 2d). Mn intoxication is postulated to inhibit Mg-dependent MST enzyme, leading to the observed accumulation of chorismate (Fig. 4b). Over-production of MST enzymes rescues YS cells (Fig. 4d), but not the PS strain, reinforcing the idea that the proximal cause of growth inhibition differs between these two phenotypically similar strains.

Our findings here extend our view of Mn homeostasis in *B. subtilis* (Fig. 5). In addition to the core MntR-regulated import (*mntH* and *mntABC*) and efflux (*mneP* and *mneS*) genes, Mn is also trafficked in the cell by two TerC family efflux proteins in support of exoenzyme metalation (28). We here demonstrate that YqgC and MnSOD are two additional factors that have protective roles in Mn homeostasis. Finally, our results emphasize that there are likely multiple ways, in which excess Mn can intoxicate cells. Even the phenotypically similar PS and YS strains appear to have distinct proximal causes for growth inhibition.

## MATERIALS AND METHODS

### Bacterial strains and growth conditions

The strains used in this study are listed in Table S1A. Mutations from *B. subtilis* 168 were moved into *B. subtilis* CU1065 as the parental WT strain for this study. Cultures were streaked from frozen glycerol stocks onto LB agar plates and grown at 37°C for 18 h. Cells were grown in 5 mL of LB broth supplemented when required with antibiotics: macrolide, lincosamide, and streptogramin (MLS: 1 μg/mL erythromycin plus 25 μg/mL lincomycin), kanamycin (15 μg/mL), and/or chloramphenicol (10 μg/mL), at 37°C under shaking conditions of 300 rpm on a gyratory shaker. Once cultures reached an OD_600nm_ ~0.4, 2 μL volume was used as an inoculum into 98 μL of LB dispensed in 96 well plates such that the initial OD_600nm_ was 0.07. These plates were supplemented with appropriate antibiotics, and IPTG/xylose was added as needed. Growth in LB or modified minimal media recipe (11) containing either glucose or malate supplemented with MnCl_2_ (Sigma) was monitored for up to 24 h at 37°C under shaking conditions using Synergy H1 (BioTek Instruments, Inc. VT) plate reader. The MIC for Mn was defined as the Mn concentration that led to an OD_600nm_ of <0.4 after 8 h of growth.

### Genetic manipulations and strain construction

#### CRISPR editing

The complementation strain was constructed using a repair template consisting of upstream (of *yqgC*) and downstream (of *sodA*) homology regions fused to *yqgC*-S936-*sodA* central fragment amplified and stitched using primers listed in the Table S1B. DNA alleles were generated by adaptor-based long-flanking homology (LFH) PCR and were subjected to stitching to generate a repair template. The repair template had compatible *SfiI* flanking the 5′ and 3′ ends. Upon digestion with *SfiI* at 50°C for 2 h, a similar restriction digest was performed for pAJS23 plasmid containing guide RNA against *erm* cassette as described previously (21). The ligation of the digested repair template into pre-digested pAJS23 was performed and was then transformed into *E. coli* DH5α selected on LB medium (Affymetrix) supplemented with 30 μg/mL of kanamycin. Plasmid was isolated and moved into *E. coli* TG1 strain. The multimeric plasmid was extracted and was then transformed into *yqgC*-S936-*sodA::erm* recipient strain grown to OD_600nm_ ~0.8 in the modified competence (MC) media. Plasmid DNA was used to transform recipient cells, which were then allowed to recover for 2 h under aerobic incubation at 30°C. Transformed *Bacillus* cells were selected on LB-kanamycin plus 0.2% mannose in the agar at 30°C (until clones appeared, i.e., ~48 h). The CRISPR plasmid was cured at 45°C for 2 days on LB agar with repeated passages for all transformants. Clones sensitive to kanamycin and erythromycin were selected, and chromosomal DNA was prepared for PCR analysis. The constructed strains were verified using Sanger sequencing of amplified PCR reactions.

#### Overexpression in pPL82 or pAX01

For ectopic expression of *Bacillus* genes (*yqgC*, *sodA*, *S936sodA*, *mneP*, *menF*, *dhbC*, and *pabB*) and heterologous expression of *Staphylococcus (mntE*) and *E. coli (yiiP/fieF*) genes, the coding regions were amplified with primers listed in Table S1B from chromosomal DNA using Phusion high-fidelity DNA polymerase (NEB) and subjected to restriction enzyme digestion, purification, and ligation into pre-digested pPL82/pAX01 vector for propagation in *E. coli* DH5α on LB supplemented with 100 μg/mL of ampicillin. Prepared recombinant plasmid constructs were transformed into recipient *Bacillus* strains for insertion at the *amyE* or *lacA* locus by double-cross over recombination selected with 10 μg/mL chloramphenicol or 1 μg/mL of erythromycin plus 25 μg/mL of lincomycin).

#### Multiple (adjacent) gene deletions

For *yqgC-S936-sodA* and *S936-sodA* deletions, upstream and downstream fragments were amplified with LFH-PCR and stitched together using joining PCR such that the donor fragment had *yqgB-erm-yqgE* (ΔYS) and *yqgC-cat-yqgE* (ΔS936-*sodA*). These fragments were then transformed into CU1065, and integrants were selected with antibiotics.

### Disk diffusion and Mn sensitivity assays

Mn sensitivity was evaluated on a MM with malate or glucose as described (11) previously. Briefly, the bottom agar contained 15 mL MMA+malate (with 1.5% final agar concentrations), and the top soft-agar consisted of 4 mL MMA+malate (with 0.75% agar) and 100 μL of mid-exponentially grown cultures (OD_600nm_ of 0.4) as inoculum. Whatman filter paper number four disks (6 mm in diameter) with Mn (10 μL of 10 mM stock) were placed on the plates, and the diameter for the zone of growth inhibition (ZOI) was measured after 18 h at 37°C.

### RRS measurements

We used DCF diacetate (DCFDA) to monitor DCF-RRS as described previously (11). Individual colonies were picked and inoculated into 5 mL LB broth (no antibiotics) and grown to an OD_600_ nm of 0.4. From these cultures, 200 µL was withdrawn and pelleted at 7,000 rpm for 2 min at room temperature, and the pellet was resuspended and washed with 400 µL 1 × liquid MM+malate (without MnCl_2_). This solution was further centrifuged, and the resultant pellet was resuspended in 200 µL 1 × liquid MM+malate. In 96-well microtiter well plate, 200 µL 1 × liquid MM+malate containing either 3 µM or 200 µM MnCl_2_ was dispensed. Two microliters of inoculum was added and cells were allowed to grow under shaking condition for 21 h at 37°C in a Synergy H1 plate reader (BioTek Instruments Inc, VT), and OD_600_ was monitored. DCFDA (Sigma) was added to a final concentration of 1.25 mg/L (5 µL of 2 mg/mL DCFDA into 200 µL), and growth was resumed with continuous shaking for another 6–8 h at 37°C, with DCF fluorescence measured using excitation and emission wavelengths of 498 and 522 nm, respectively. DCF-reactive RRS levels were inferred from the stable level achieved within the first 60 min. incubation and calculated as fluorescence intensity divided by OD_600_ at the time of DCFDA addition.

### Real-time RT-PCR

Total RNA was extracted using a QIAGEN kit from 1.5 mL of mid-log (OD_600nm_ = 0.4) WT and YS cells, which were grown in LB broth either in the presence or absence of 150 μM Mn. Total RNA was treated with DNase (Ambion) enzyme to further purify and remove traces of DNA. For each reaction, 2 µg of RNA was used for cDNA preparation using a high-capacity reverse transcriptase (Applied Biosystems) kit amplified with random hexamer primers. Further, for amplicon measurements 10 ng of cDNA was used as a template along with 500 nM of *mneP*, *mneS*, and *gyrA* (control) gene-specific qPCR F/R primers in a 1X SYBR green master mix (Bio-Rad). Threshold and baseline parameters were kept consistent for experiments performed on different days. All C_t_ mean values for the gene expression were normalized to *gyrA* (*n* = 3).

### mTn mutagenesis

The YS cells containing pMarA plasmid were grown in LB broth with erythromycin (1 µg/mL) at 30°C till OD_600_ reached ~1.0, and cells were plated onto Mn gradient plate made up of MM containing glucose at 42°C. Colonies that grew after overnight incubation near the high levels of Mn were sub-cultured in LB supplemented with kanamycin (15 µg/mL) at 37°C to confirm the presence of mTn. Chromosomal DNA was purified from these DYS-Tn cells and was subjected to *TaqαΙ* restriction enzyme digestion at 37°C for 2 h, followed by overnight ligation of cohesive ends to generate a circular transposon-gDNA chimeric library. The PCR reaction from ends of the mTn performed on the chimeric library was further subjected to Sanger’ sequencing analysis at Cornell biotechnology resource center (BRC) facility to identify the mTn insertion site within the genome (Table S1D).

### Whole genome re-sequencing

Suppressors for YS strains were isolated using Mn-disk diffusion assay performed using either LB- with 150 μM Mn present in the broth, 100 μM Mn supplemented in a plate condition, or a disk diffusion performed in a MM-malate agar. For MMA+malate, suppressors were picked from the clearance zone. All the suppressors were tested multiple times for the increased resistance to Mn and genomic DNA was extracted, purified, and subjected to Illumina sequencing (San Diego, CA) by MiGS or SeqCenter (Pittsburgh). The Illumina sequence reads were trimmed, mapped, aligned, and analyzed for sequence variants against the reference genome sequence NC_000964 using CLC genomics workbench software (Table S1C). The nucleotide changes (relative to reference) found in both the parental WT (CU1065) and the YS (unstressed) strains were ignored, and only newly arising nucleotide variants found in the suppressor strains are indicated.

### Determination of intracellular metal content

ICP-ES analysis was performed using modifications of a previously described procedure (11). Briefly, strains were grown in 5 LB broth to an OD_600nm_ of 0.4 at 37°C under aerobic growth conditions. Cultures were treated with or without 150 µM Mn and further grown for 30 min. 2 mL of samples were collected by centrifugation and washed twice with 5 mL Chelex-treated phosphate-buffered saline (PBS), resuspended in 0.5 mL PBS, and weighed. Samples were digested in double distilled HNO_3_ in a carbon heat block using a Vulcan 84 automated sample digestion unit (http://www.qtechcorp.com/), followed by the addition of 60/40 nitric/perchloric acid and incubation at 150°C. Samples were cooled down brought to a constant final volume with deionized 18 MOhm water with 5% HNO_3_. ICP analysis was run on an Agilent 7700 series ICP-ES housed at the US Department of Agriculture—ARS Robert W. Holley Center for Agriculture and Health in Ithaca, NY [mean ± standard deviation (SD); *n* = 3 independent biological replicates, each containing three technical replicates].

### β-galactosidase plates and enzyme activity

All bacterial cultures were grown to a mid-log phase, streaked onto LB agar containing 100 mg/mL of X-gal and Mn (0–50 μM), and plates were incubated at 37°C overnight. Blue color development for different strains was noted by capturing images.

### Metabolite extraction and measurements

All strains were grown in LB at 37°C until the culture reached an OD_600_ of 0.4 and then treated with or without 0.15 mM (final concentration) of MnCl_2_ (Sigma-Aldrich) for 60 min at 37°C. Cells were pelleted and quenched by resuspending in chilled 0.6 mL of mixtures of acetonitrile:methanol:water (40:40:20). These were further lysed using 0.1 mm Zirconia beads in a Precellys homogenizer (Bertin Instruments). Freshly lysed suspensions were centrifuged at ~12,000 rpm for 8 min at 37°C. The supernatants were passed through a 0.22 mm SpinX tube filter (Sigma-Aldrich). Extracted metabolites were separated on a Cogent Diamond Hydride Type C column of 1200 liquid chromatography coupled to an Mass 6220 time-of-fllight (TOF) spectrophotometer (Agilent). Ion abundances of metabolites were estimated using Profinder 8.0 and log_2_ fold changes were analyzed and plotted using Gene Cluster 3.0 and Java Treeview.

### Quantification of siderophores

*B. subtilis* cultures were aerobically grown overnight in MM-malate (1 μM of Fe) at 37°C with and without 100 μM added Mn. Optical density at 600 nm was noted, and cells were harvested by centrifugation at 5,000 rpm for 10 min to retrieve supernatant, and to 1 mL supernatant 200 μL of 10 mM FeCl_3_ (Sigma) dissolved in 100 mM HCl was added. This supernatant fraction was then neutralized by the addition of 100 μL of 1 M Tris-HCl (pH 8.0), and the absorbance at 510 nm was recorded. A standard curve was prepared with DHBA (Sigma) under similar conditions to extrapolate levels of siderophores in unknown culture samples. All 510 nm values were normalized to culture OD at 600 nm (*n* = 3). Color development for different reaction tubes was captured using an iPhone XS (Apple, CA).

## Data Availability

The metabolomics data sets generated during the current study are available in the Metabolomics Workbench (48) repository under accession number PR001779 and DOI http://dx.doi.org/10.21228/M8RM7K. All other data supporting the findings of this study are available within the paper and its supplemental files.

## SUPPLEMENTAL MATERIAL

Supplemental figures jb.00052-24-s0001.pdf Fig. S1 to S4.

Supplemental tables jb.00052-24-s0002.xlsx Tables S1A to D.

ASM does not own the copyrights to Supplemental Material that may be linked to, or accessed through, an article. The authors have granted ASM a non-exclusive, world-wide license to publish the Supplemental Material files. Please contact the corresponding author directly for reuse.
