# Supplementary material for: The Bacillus subtilis yqgC-sodA operon protects magnesium-dependent enzymes by supporting manganese efflux
Source: J Bacteriol. 2024 May 31;206(6):e00052-24. doi: 10.1128/jb.00052-24 (PMC11332163; doi:10.1128/jb.00052-24)
Supplement: Supplemental figures — Fig. S1 to S4. [file jb.00052-24-s0001.pdf]

Supplementary Material

The *Bacillus subtilis yqgC-sodA* operon protects magnesium-dependent enzymes by supporting manganese efflux

Ankita J. Sachla*[1], Vijay Soni[2], Miguel Piñeros[3,4], Yuanchan Luo[1,5], Janice J. Im[1], Kyu Y. Rhee[2], John D. Helmann[1]*

[1]Cornell University, Department of Microbiology, Ithaca, NY, 14853-8101, USA.
[2]Division of Infectious Diseases, Weill Department of Medicine, Weill Cornell Medicine, New York, NY 10065, USA.
[3] School of Integrative Plant Sciences, Plant Biology Section, Cornell University, Ithaca, NY 14853, USA
[4] Robert W. Holley Center for Agriculture and Health, USDA-ARS, Ithaca, NY 14853, USA
[5]State Key Laboratory of Bioreactor Engineering, East China University of Science and Technology, Shanghai, China.

*corresponding authors: jdh9@cornell.edu; ajs588@cornell.edu

List of the supplementary figures

Fig. S1: Growth of various strains in MM+malate with variable Mn.

Fig S2: Growth of various strains in the presence of paraquat.

Fig S3: Metabolite profile of various strains with and without Mn stress.

Fig S4: Image illustrating DHBA (siderophore) production across different strains.

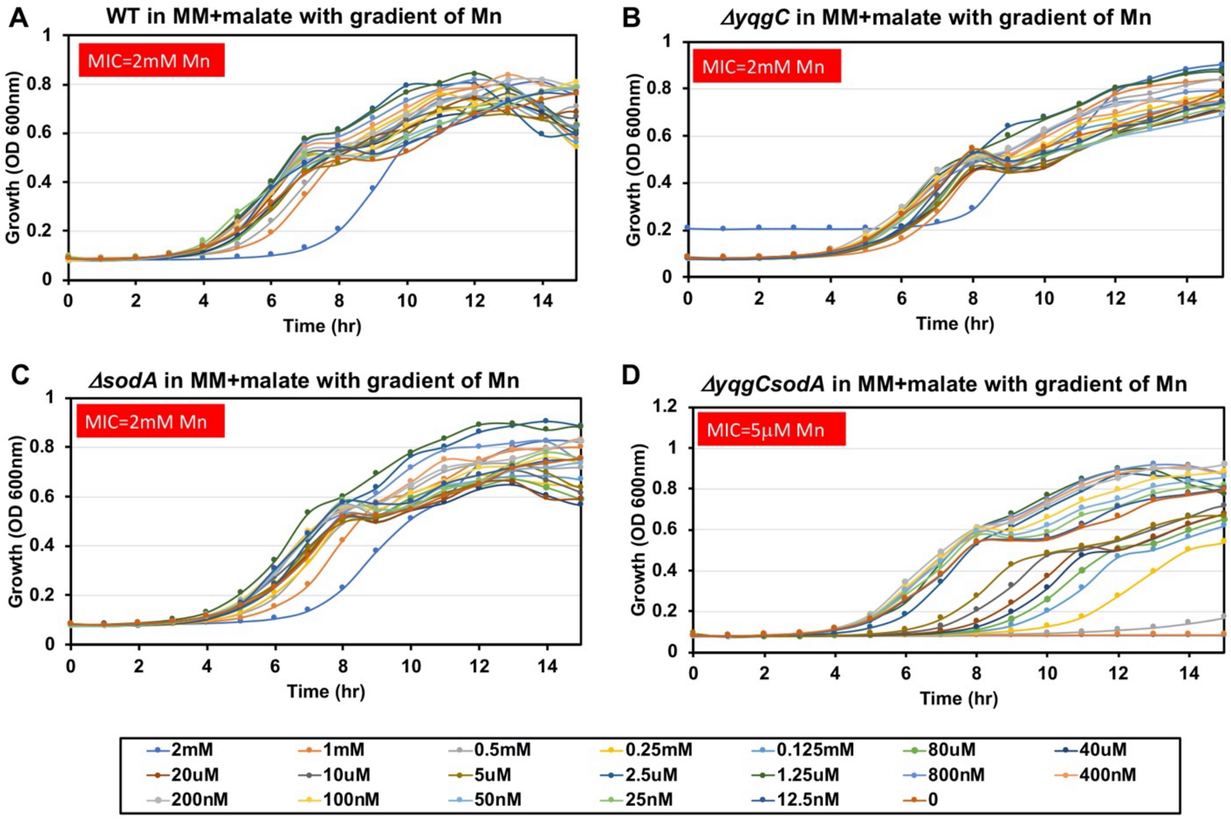

Fig. S1: Growth of various strains in MM+malate with variable Mn. Time resolved OD_{600nm} of cultures: A) WT, B) *yqgC* deletion, C) *sodA* deletion, and D) *yqgC-S936-sodA* operon deletion monitored aerobically at 37 °C for 18 hr. with 0-2 mM of Mn amendment. The Mn MIC was determined as the concentration at which cultures showed less than 0.4 OD_{600nm} at 8 hr (50% inhibition).

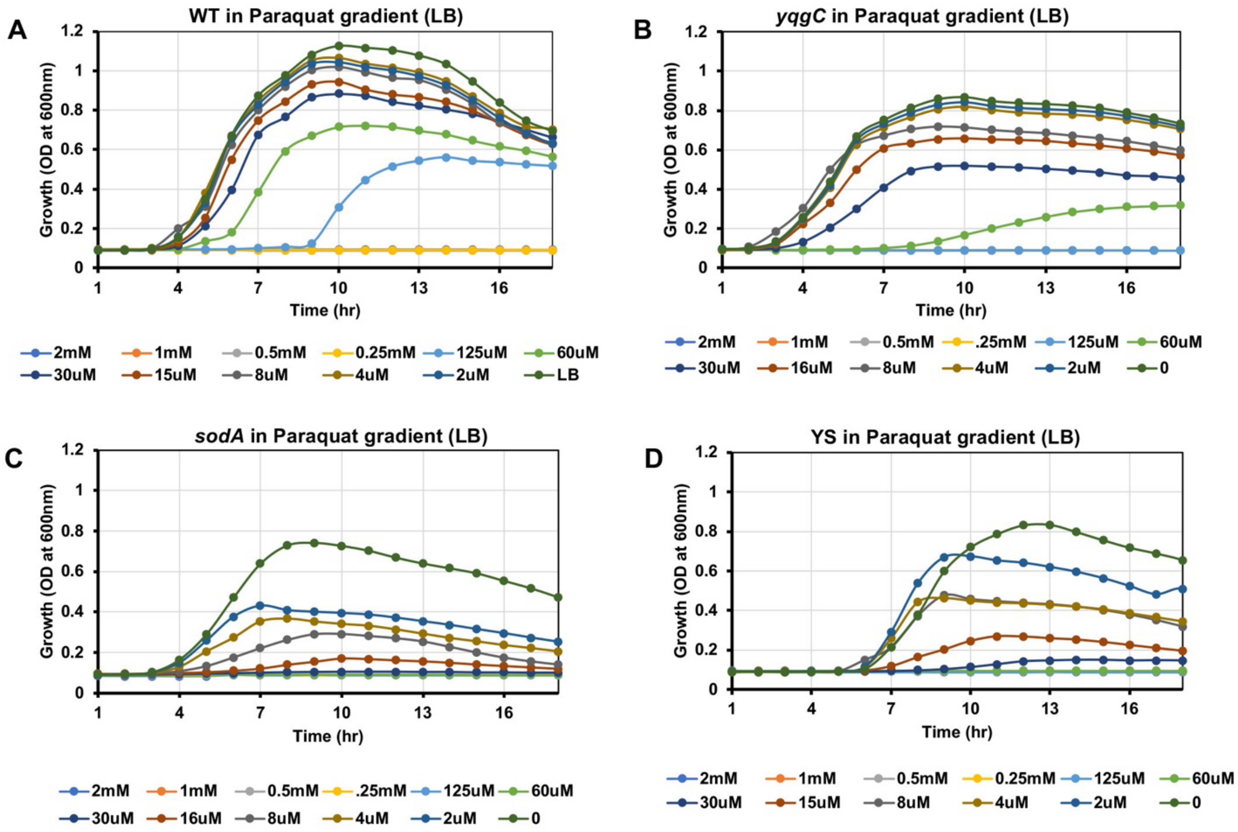

Fig. S2: Growth of various strains in the presence of Paraquat. Time resolved OD_{600nm} of cultures: A) WT, B) *yqgC* deletion, C) *sodA* deletion, and D) *yqgC-S936-sodA* operon deletion was monitored aerobically at 37 °C for 18 hr. During growth measurements 0-2 mM of paraquat were added. Paraquat MIC was determined as a concentration at which cultures showed less than 0.4 OD_{600nm} at 8 hr (50% inhibition). WT=60 μM, *yqgC*=30 μM, *sodA*=4 μM, YS=4 μM. Note: unusual lag time for YS strains.

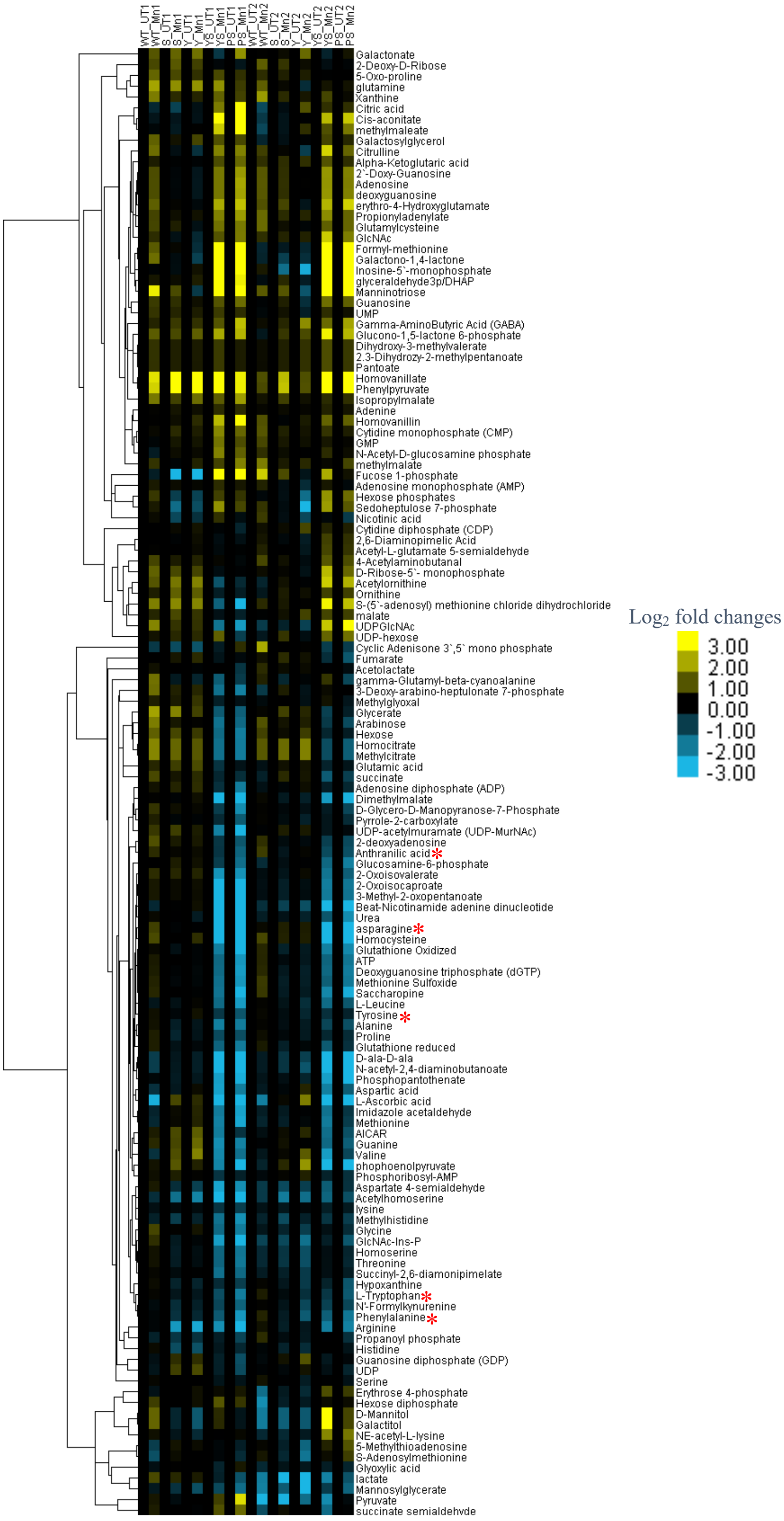
WT_UT1
WT_Mn1
S_UT1
S_Mn1
Y_UT1
Y_Mn1
YS_UT1
YS_Mn1
PS_UT1
PS_Mn1
WT_UT2
WT_Mn2
S_UT2
S_Mn2
Y_UT2
Y_Mn2
YS_UT2
YS_Mn2
PS_UT2
PS_Mn2
Galactonate
2-Deoxy-D-Ribose
5-Oxo-proline
glutamine
Xanthine
Citric acid
Cis-aconitate
methylmaleate
Galactosylglycerol
Citrulline
Alpha-Ketoglutaric acid
2`-Doxy-Guanosine
Adenosine
deoxyguanosine
erythro-4-Hydroxyglutamate
Propionyladenylate
Glutamylcysteine
GlcNAc
Formyl-methionine
Galactono-1,4-lactone
Inosine-5`-monophosphate
glyceraldehyde3p/DHAP
Manninotriose
Guanosine
UMP
Gamma-AminoButyric Acid (GABA)
Glucono-1,5-lactone 6-phosphate
Dihydroxy-3-methylvalerate
2.3-Dihydrozy-2-methylpentanoate
Pantoate
Homovanillate
Phenylpyruvate
Isopropylmalate
Adenine
Homovanillin
Cytidine monophosphate (CMP)
GMP
N-Acetyl-D-glucosamine phosphate
methylmalate
Fucose 1-phosphate
Adenosine monophosphate (AMP)
Hexose phosphates
Sedoheptulose 7-phosphate
Nicotinic acid
Cytidine diphosphate (CDP)
2,6-Diaminopimelic Acid
Acetyl-L-glutamate 5-semialdehyde
4-Acetylaminobutanal
D-Ribose-5`- monophosphate
Acetylornithine
Ornithine
S-(5`-adenosyl) methionine chloride dihydrochloride
malate
UDPGlcNAc
UDP-hexose
Cyclic Adenisone 3`,5` mono phosphate
Fumarate
Acetolactate
gamma-Glutamyl-beta-cyanoalanine
3-Deoxy-arabino-heptulonate 7-phosphate
Methylglyoxal
Glycerate
Arabinose
Hexose
Homocitrate
Methylcitrate
Glutamic acid
succinate
Adenosine diphosphate (ADP)
Dimethylmalate
D-Glycero-D-Manopyranose-7-Phosphate
Pyrrole-2-carboxylate
UDP-acetylmuramate (UDP-MurNAc)
2-deoxyadenosine
Anthranilic acid *
Glucosamine-6-phosphate
2-Oxoisovalerate
2-Oxoisocaproate
3-Methyl-2-oxopentanoate
Beat-Nicotinamide adenine dinucleotide
Urea
asparagine *
Homocysteine
Glutathione Oxidized
ATP
Deoxyguanosine triphosphate (dGTP)
Methionine Sulfoxide
Saccharopine
L-Leucine
Tyrosine *
Alanine
Proline
Glutathione reduced
D-ala-D-ala
N-acetyl-2,4-diaminobutanoate
Phosphopantothenate
Aspartic acid
L-Ascorbic acid
Imidazole acetaldehyde
Methionine
AICAR
Guanine
Valine
phophoenolpyruvate
Phosphoribosyl-AMP
Aspartate 4-semialdehyde
Acetylhomoserine
lysine
Methylhistidine
Glycine
GlcNAc-Ins-P
Homoserine
Threonine
Succinyl-2,6-diamonipimelate
Hypoxanthine
L-Tryptophan *
N'-Formylkynurenine
Phenylalanine *
Arginine
Propanoyl phosphate
Histidine
Guanosine diphosphate (GDP)
UDP
Serine
Erythrose 4-phosphate
Hexose diphosphate
D-Mannitol
Galactitol
NE-acetyl-L-lysine
5-Methylthioadenosine
S-Adenosylmethionine
Glyoxylic acid
lactate
Mannosylglycerate
Pyruvate
succinate semialdehyde
Log2 fold changes
3.00
2.00
1.00
0.00
-1.00
-2.00
-3.00

Fig S3: Metabolic profile of various strains with and without Mn stress.

Heatmap representing log_2 fold changes of metabolites in Mn-treated cells (Mn) as compared to untreated (UT) strains. Columns depict bacterial strains with or without Mn, starting with WT followed by *sodA* (S), *yqgC* (Y), *yqgC-sodA* (YS), and *mneP mneS* (PS) from two independent experiments (1 and 2). Rows show the log_2 fold changes of individual metabolites. Data were analyzed using Gene Cluster 3.0 software with uncentered Pearson's correlation and centroid clustering methods. Image was generated using Java Treeview. The red * indicate metabolites downregulated in excess Mn and mentioned in the main text.

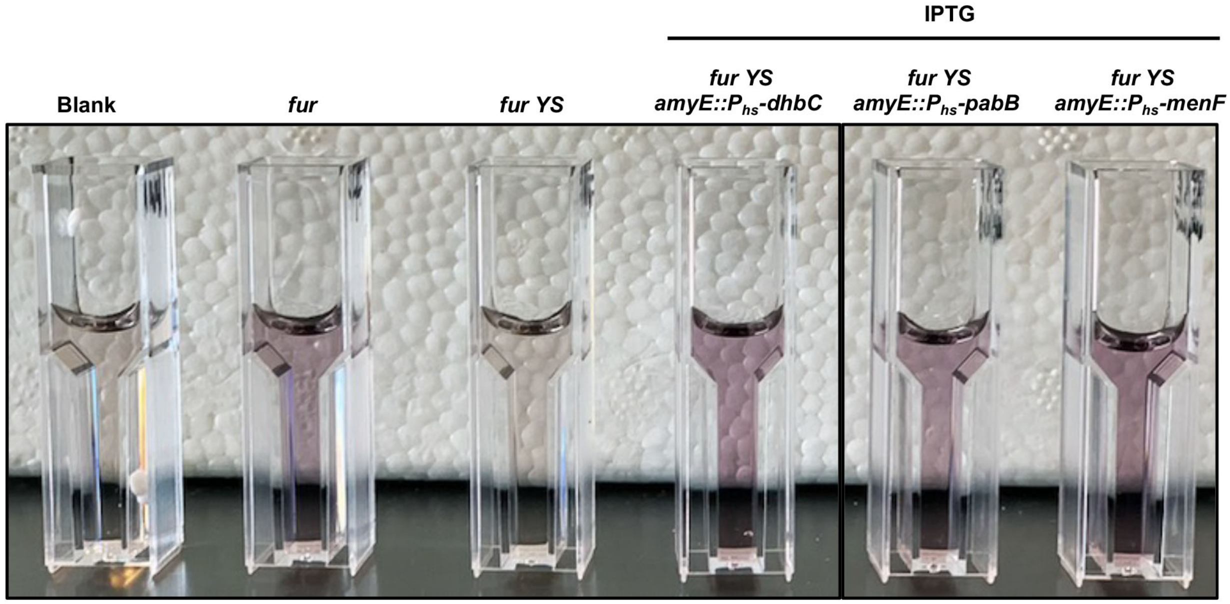

Fig S4: Image illustrating DHBA(G) (siderophore) production across different strains. Various strains were grown aerobically at 37 °C for 18 hr in MMM containing limited amount of iron and supernatants were collected and were supplied with excess of exogenous iron following neutralization with buffer. The intensity of the magenta color is proportional to the DHBA(G) levels secreted by the cells during overnight growth with iron limitation.
